# High-Performance Flexible Solid-State Carbon Cloth Supercapacitors Based on Highly Processible N-Graphene Doped Polyacrylic Acid/Polyaniline Composites

**DOI:** 10.1038/srep12883

**Published:** 2016-02-17

**Authors:** Yongguang Wang, Shaochun Tang, Sascha Vongehr, Junaid Ali Syed, Xiangyu Wang, Xiangkang Meng

**Affiliations:** 1 Institute of Materials Engineering, National Laboratory of Solid State Microstructures, and College of Engineering Applied Sciences, Nanjing University, Jiangsu, P. R. China

## Abstract

Improving the solubility of conductive polymers to facilitate processing usually decreases their conductivity, and they suffer from poor cycling stability due to swelling-shrinking during charging cycles. We circumvent these problems with a novel preparation method for nitrogen-doped graphene (NG) enhanced polyacrylic acid/polyaniline (NG-PAA/PANI) composites, ensuring excellent processibility for scalable production. The content of PANI is maximized under the constraint of still allowing defect-free coatings on filaments of carbon cloth (CC). The NG content is then adjusted to optimize specific capacitance. The optimal CC electrodes have 32 wt.% PANI and 1.3 wt.% NG, thus achieving a high capacitance of 521 F/g at 0.5 F/g. A symmetric supercapacitor made from 20 wt.% PANI CC electrodes has more than four times the capacitance (68 F/g at 1 A/g) of previously reported flexible capacitors based on PANI-carbon nanotube composites, and it retains the full capacitance under large bending angles. The capacitor exhibits high energy and power densities (5.8 Wh/kg at 1.1 kW/kg), a superior rate capability (still 81% of the 1 A/g capacitance at 10 A/g), and long-term electrochemical stability (83.2% retention after 2000 cycles).

Recent developments in portable and wearable/foldable electronics have increased the demand for high performance energy storage systems that are required to be small, lightweight, and able to endure large deformations in long-term applications^1^,^2^,^3^. Flexible all-solid-state supercapacitors are very promising regarding most of these requirements^4^,^5^,^6^, but they usually have lower energy densities (≦10 W h kg^−1^) than rechargeable batteries^3^. The key to achieve higher energy densities is improving the electrode materials on the nanoscale. Among the currently favored electrode materials, pseudo-capacitive conducting polymers are the easiest processed and flexible^7^, and their composition, film thickness, and nano architecture are well controllable. However, they usually do not solve in water and therefore obtain powders from water based synthesis. Electrodeposition leads to brittle films^8^,^9^,^10^, which limits practical applications. Moreover, they usually suffer the serious disadvantage of poor cycling stability as the redox sites in the polymer backbone are not sufficiently stable to survive the swelling-shrinking over many charging cycles^11^.

Polyaniline (PANI) is generally considered to be one of the most intriguing materials for supercapacitors due to its relatively high conductivity and specific capacitance, low cost, and convenient preparation^12^,^13^. However, the common highly conducting form of PANI, namely protonic acid doped PANI, has poor solubility in water, making processing very difficult. In order to increase the solubility of PANI, various strategies like doping with macromolecular acids^14^ and introduction of substituent groups^15^ have been pursued, but homogeneous composites as well as continuous, defect-free and highly flexible coverings are difficult to achieve. One strategy is to embed PANI in a polymer host matrix like polyvinyl carbazole^16^, polymethyl methacrylate^17^, poly(vinyl alcohol)^18^, or polycarbonate^19^, but these matrixes are insulators. Generally, the electroactivity of composites increases with the conductivity, but the solubility then decreases again^20^. Inclusion of inorganic nanoparticles (NPs) like carbon nanotubes into conducting polymers is an effective way to enhance both the mechanical and electrochemical properties. In particular, addition of low concentrations (~0.05–5 wt%) of graphene oxide (GO) and graphene-based NPs into polymers enhances conductivity and mechanical properties exceptionally well. Graphene consists of single-atom layers and has thus naturally a large specific surface area^21^. Nitrogen-doping, due to the incorporation of the nitrogen as a heteroatom, improves graphene’s high conductivity even further^22^. N-doped graphene (NG) is therefore very promising for supercapacitor electrode materials^23^,^24^,^25^,^26^.

Carbon cloth (CC) is an inexpensive, conducting textile and thus an excellent current collector as well as high surface area support. It is highly flexible yet also has excellent strength^27^,^28^,^29^. The large pores of the cloth together with the smaller micro and nanopores of the added polymer coverings result in a hierarchical, almost scale invariant pore distribution that facilitates the diffusion of electrolyte into the electrode material^30^. The present work reports a novel method for preparing NG-doped polyacrylic acid/polyaniline (NG-PAA/PANI) composites obtaining excellent processibility for defect-free, highly flexible carbon fiber coatings, ensuring stable electrochemical properties. Interestingly, we combine two steps (see Fig. 1) that both, if taken in isolation as a strategy, worsen solubility. PANI is supplied by both, the first and second step, this separation into two steps being crucial. The content of PANI can be maximized to 32% under the constraint of still allowing defect-free coatings on single filaments of CC, and the NG content is then adjusted to optimize specific capacitance. Symmetric all-solid-state supercapacitors made from two CC electrodes impregnated with NG-PAA/PANI having only 20% PANI already reach a capacitance of 68 F/g at 1 A/g, which is 13 times larger than previously reported capacitors based on a similar PAA/PANI composite^24^, and also more than four times larger than reported flexible capacitors with PANI-carbon nanotube composites^31^.

## Results and Discussion

**Figure 1:**
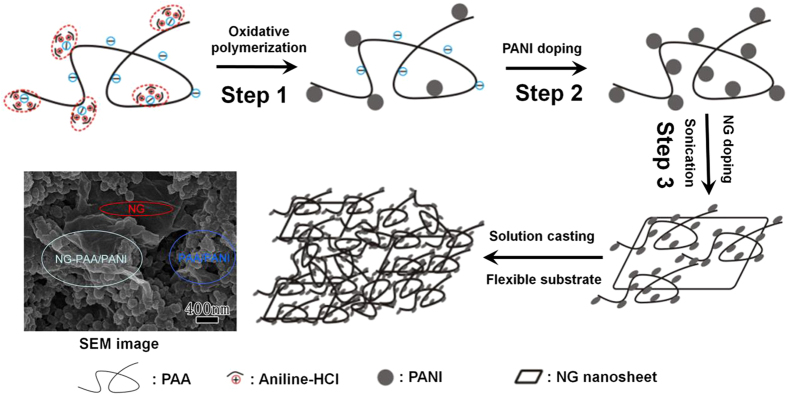
Illustration of the process from synthesis to obtaining a NG-PAA/PANI composite coating on CC; PANI is supplied by both, the first and second step. NG doping in step 3 improves the conductivity further and reduces swelling.

The whole procedure from synthesis of NG-PAA/PANI composites to obtaining covered CC is illustrated in Fig. 1. We combine two steps that both in isolation worsen solubility. Step 1 is an *in-situ* polymerization obtaining PAA/PANI copolymer in a stable, water based solution. Step 2 is the addition of poorly water soluble PANI NPs. A homogeneous suspension with a high PANI content that can be processed into defect-free fiber coverings cannot be obtained via increasing PANI with the first or the second step alone. Combining the two steps works, because the PAA/PANI copolymer helps stabilizing the PANI NPs in suspension. We maximize the addition of NPs under the constraint of still allowing defect free covers around the CC fibers. NG doping (Step 3) reduces the swelling/contracting of PANI during recharging cycles, and it increases electron transport between the particles similar to carbon nanotube doping^32^,^33^. Table 1 presents the used concentration of PAA (*C*_PAA_) and the resulting PANI content in the solution according to UV-Vis absorption^34^, versus specific capacitance and conductivity of the PANI-PAA composite films, all using PAA/PANI solutions obtained only from the polymerization (first step, before addition of PANI NP and NG). Capacitance and conductivity both decrease with *C*_PAA_, but stable solutions cannot be obtained below 0.02 mM, where precipitation is visible after about 2 h. We therefore chose *C*_PAA_ = 0.02 mM, which makes the PANI content ~1 wt.% (Fig. S1).

**Figure 2:**
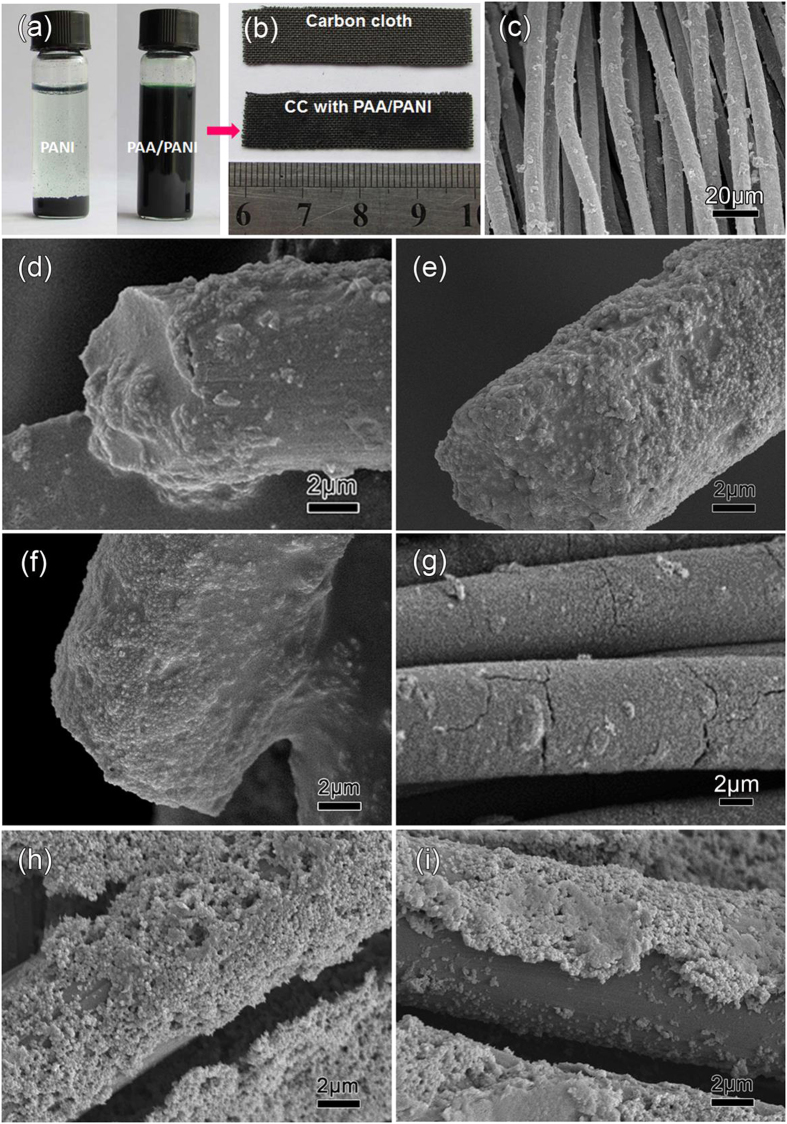
(**a**) Photographs of the aqueous solutions containing pure PANI and PAA/PANI composite with 69 wt% PANI, (**b**) a CC substrate before and after impregnation with a PAA/PANI suspension containing 32 wt.% PANI, and (**c**) its low-magnification SEM. Magnified SEM images of single carbon fibers coated by PAA/PANI with PANI contents of 10, 20, 32, 40, 45 and 56 wt.% (d to i, respectively).

Figure 2a shows an aqueous solution containing pure PANI NPs, obviously precipitated in the left bottle, and the same particles added into the PAA/PANI solution (right bottle), which obtains a homogeneous suspension that is stable for several months. While the PANI percentage could not be increased above 1 wt.% in the first step above, the stable suspensions after subsequent addition of PANI NPs can contain up to 69 wt.% PANI. Using larger aniline concentrations too early produces more PANI in the polymerization, leading to large particles that precipitate. In the two-step method, the added PANI NPs attach to the carboxylic functional groups of the PAA^35^. The resulting PANI particles stay suspended because of the long chains that are attached to them.

High performance flexible devices strongly depend on the mechanical integrity of their materials. Figure 2b shows a CC substrate before and after impregnation with a PAA/PANI suspension containing 32 wt.% PANI; the dried cloth is darker after impregnation. As can be seen in a low-magnification SEM image (Fig. 2c), individual CC filaments are covered uniformly by the PAA/PANI coating. Single carbon fibers coated by PAA/PANI with different weight percentages of PANI (*w*_PANI_, per dried film) are shown in Fig. 2d–i. Below 32 wt.%, the PAA/PANI coats are continuous and uniform (Fig. 2d–f). Carbon and nitrogen elemental mappings (Fig. S2) confirm that of a single CC fiber is covered homogeneously by PANI NPs. At 40 wt.%, some cracks form (Fig. 2g). Increasing *w*_PANI_ to 45 wt.% resulted in uncovered areas next to thick aggregates (Fig. 2h). There is too little PAA/PANI matrix per added PANI NPs. With less macromolecular matrix holding the film together against the slight contraction due to drying, the films break up. This will later lead to delamination of the coating under repeated charging (see Fig. S3) or bending. As *w*_PANI_ was further increased to 56 wt.%, the coatings already peel off from the surface of the fibers (Fig. 2i).

**Figure 3:**
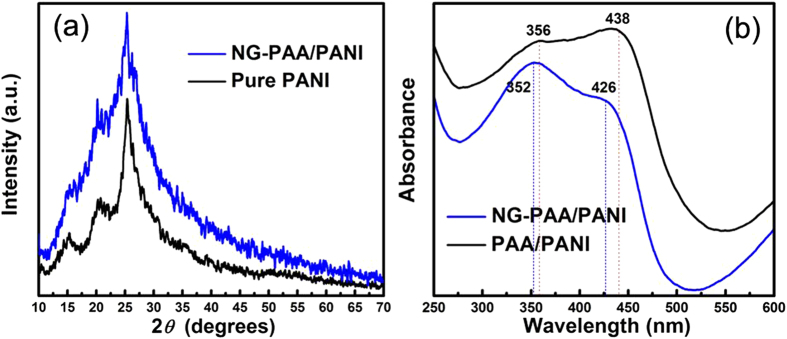
(**a**) XRD patterns of pure PANI and the NG-PAA/PANI composite (32 wt.% PANI, 1.3 wt.% NG), and (**b**) UV-Vis absorption of PAA/PANI (*w*_PANI_ = 32 wt.%) and NG-PAA/PANI composites (shifted along y-axis to facilitate comparison of peak positions).

In order to dope the PAA/PANI composite with NG (before coating on CC), hydrothermal reduction with the organic reducer ethylenediamine was used so that some oxygen species such as hydroxyl, carbonyl, and carboxyl remain while nitrogen species are introduced. The remaining oxygen groups can promote the dispersion and combination of NG sheets with PAA/PANI composites^36^,^37^. XRD (Fig. S4a), FT-IR (Fig. S4b), and XPS (Fig. S4c,d) analyses confirm a complete conversion of GO into NG. Figure 3a compares XRD patterns of pure PANI and the NG-PAA/PANI composite (*w*_PANI_ = 20 wt.%, *w*_G_ = 1.3 wt.%). The diffraction peaks at 2*θ* = 16.34°, 20.41° and 25.32° correspond to PANI. The broad amorphous band peaked at 18.42° is due to the PAA. The PANI related peaks are not shifted in the NG-PAA/PANI spectrum, which indicates that no other organics were generated through the PAA action on PANI. In UV-visible absorption spectra (Fig. 3b), the PAA/PANI composites show two peaks at 356 nm and 438 nm due to *π*–*π* and polaronic transition^38^. NG doping shifts both peaks towards shorter wavelengths, confirming a chemical interaction between NG and PAA/PANI. The -COOH groups on the NG are hydrogen bonded with the -NH group of PANI.

**Figure 4:**
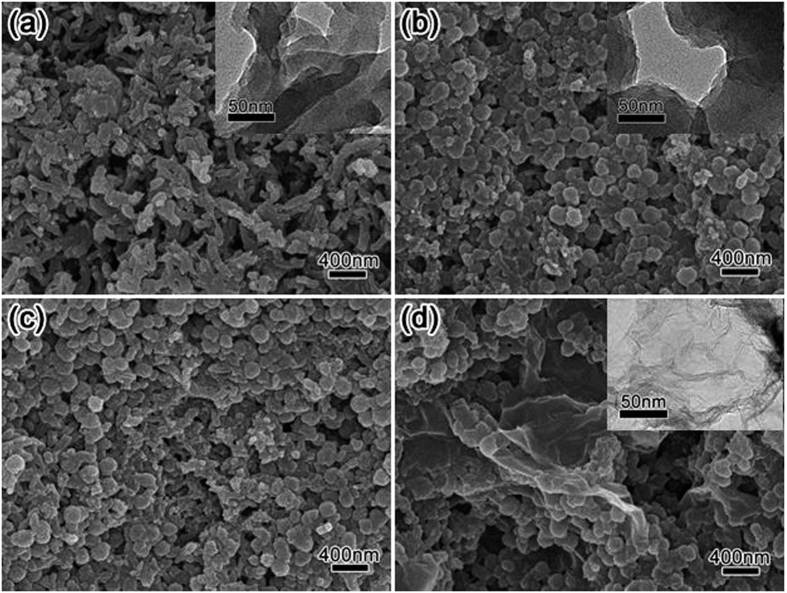
SEM images of (**a**) pure PANI, (**b**) only *in-situ* polymerization PAA-PANI (no added PANI nanoparticles), (**c**) PAA/PANI (after PANI NPs doping), and (**d**) NG-PAA/PANI composites. The insets are high-magnification TEM images.

The irregular morphology of the pure PANI particles (Fig. 4a) distinguishes them from the spherical shape of the *in-situ* polymerization PAA/PANI particles (Fig. 4b). After addition of PANI particles, a mixture of both morphologies is obtained (Fig. 4c), which shows that most of the PAA/PANI agglomerated just as it does without added PANI NPs (meaning for example that the PAA chains did not all wrap around the irregular PANI for example^39^). The presence of the irregular, often concave PANI shapes contributes to porosity. Most of the known PANI composites doped with insulating polymers such as PAA show two distinguishable phases^40^. The here described method results in a very well mixed, single phase. Figure 4d shows how also the NG integrates closely with the PAA/PANI. According to the weight percentage, little NG was added, but NG is very light and the NG sheets are also very thin (see TEM image in the inset). NG can therefore be found all over the sample and the sheets spread out far to connect many PAA/PANI nanoparticles, greatly improving electron transfer.

**Figure 5:**
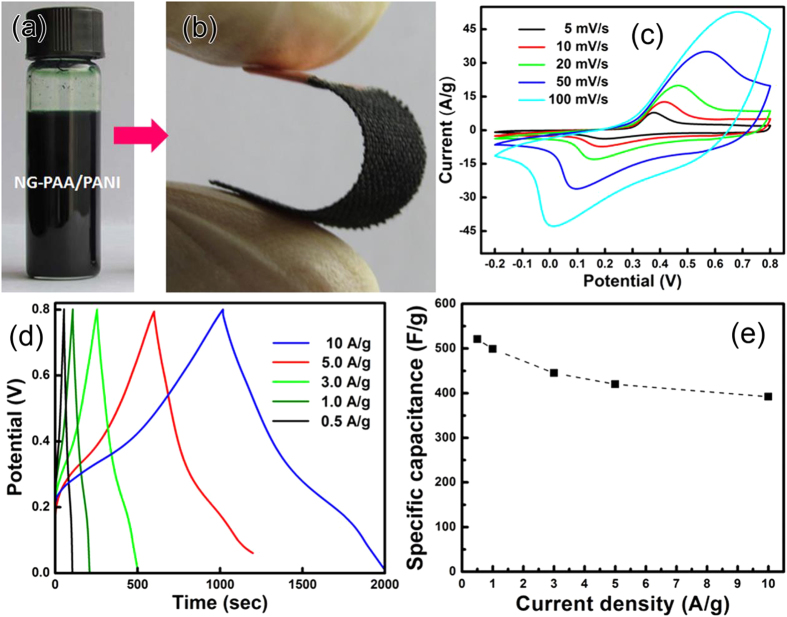
Photographs of (**a**) the aqueous NG-PAA/PANI suspension containing 32 wt.% PANI and 1.3 wt.% NG, (**b**) a single bent CC electrode. (**c**) CV curves at different scan rates, and (**d**) Galvanostatic charge/discharge curves at different current densities of the optimal NG-PAA/PANI on CC in 1 M H_2_SO_4_, and (**e**) the corresponding specific capacitance vs. current density.

The NG-PAA/PANI dispersion is also homogeneous and stable (see Fig. 5a). In order to optimize NG content, we investigated the electrochemical properties of NG-PAA/PANI composite films with different NG percentages. The capacitance is optimal at 1.3 wt.% (Fig. S5a), which is already higher than that of pure PANI nanoparticles (~150 F/g)^41^,^42^, which of course has 100 wt.% PANI. Thus, PAA does not only serve to disperse more PANI in the suspension. It also improves the desired electrochemical properties, similar to the studies that dope PANI with PAA^20^. The improvement can be partially attributed to the modification of PANI by the interaction with the PAA chains through acid base reaction^43^. As usually observed with graphene doping^39^, the performance increases steeply as soon as NG is added, which is consistent with that it facilitates electron transport. Further addition beyond the maximum where optimal conduction is ensured will merely decrease the content of electrochemically active material.

We measured CV curves of CC electrodes with the optimal NG-PAA/PANI composite (32 wt.% PANI, 1.3 wt.% NG). The area of the curves increases with the scan rate, and the curves’ shape reveals mainly Faradic pseudo-capacitance (Fig. 5c). Since the CC itself is somewhat active as an electrode material for supercapacitors, the typical redox and reduction peaks for PANI^10^ are not observable in the CV curves. However, pure CC has a low specific capacitance (2 mF/cm^2^ from the CV curve in Fig. S5b), consistent with the literature (1–2 F/g)^44^, which means that the capacitance of impregnated CC electrodes is mainly due to the NG-PAA/PANI composites. Galvanostatic CD curves at different current densities (Fig. 5d) show almost no potential drops after the peak. The corresponding specific capacitances are 521, 499, 445, 420, and 392 F g^−1^ at current densities of 0.5, 1.0, 3.0, 5.0 and 10 A g^−1^ (Fig. 5e). The capacitance of 521 F/g at 0.5 A g^−1^ locates in the upper range of the reported values for flexible polymer films^12^. Compared with paper-like composite films of G-PANI-nanofibers^45^ with 210 F/g at 1.0 A g^−1^, our composite more than doubles performance. The CC electrode still has 392 F/g even at 10 A g^−1^ (75% of the capacitance at 0.5 A g^−1^), making it very promising for rapid charge/discharge applications.

**Figure 6:**
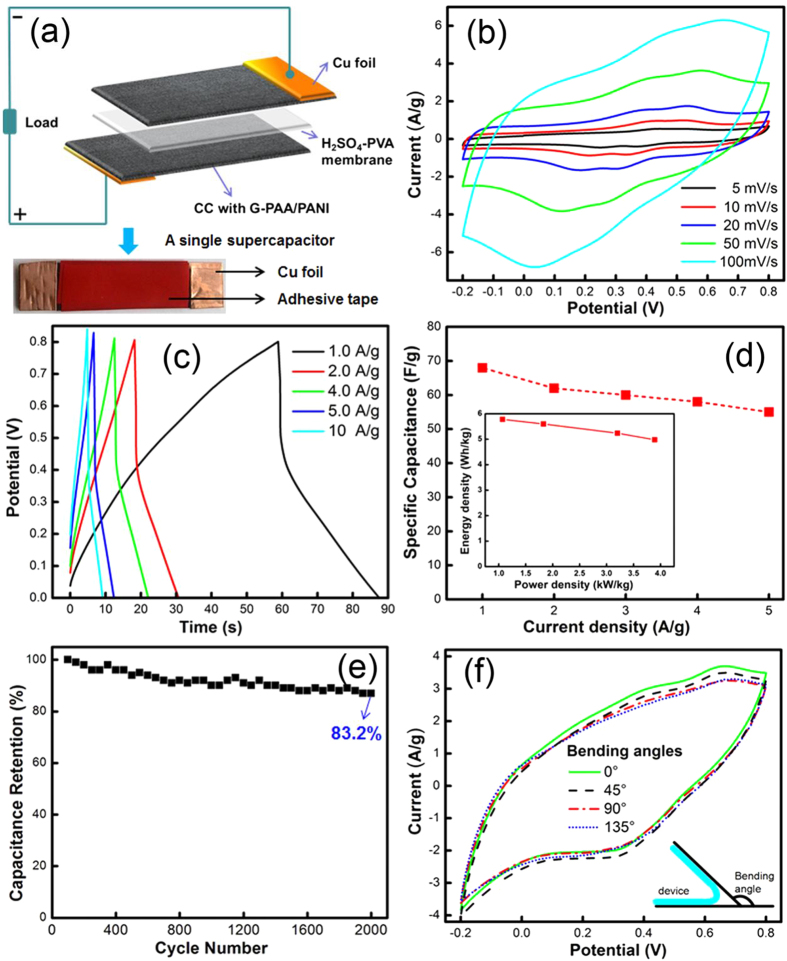
(**a**) Illustration of the assembly of the all-solid-state capacitor (optical image below), and (**b**) its CV curves at different scan rates and (**c**) Galvanostatic CD curves at different current densities, (**d**) specific capacitances at different current densities (the inset shows the energy density vs. power density), (**e**) cycling performance of the all-solid-state capacitor based on the optimal NG-PAA/PANI at 1.0 A/g over 2000 cycles, and (**f**) CV curves at a scan rate of 50 mV/s for the capacitor under different bending angles.

Although there are many reports about PANI based composite electrode materials, reports of flexible supercapacitors assembled with such composites are limited^7^,^20^,^31^,^46^. Figure 6a illustrates the sandwich structure of a flexible all-solid-state supercapacitor assembled from two impregnated CC with H_2_SO_4_-PVA electrolyte in between; a photo is shown below (the investigated capacitor has a 20% PANI composite). The CV curves (Fig. 6b) are much more rectangular now than for the electrodes (Fig. 5c), indicating a contribution from double-layer capacitance. Charging at a current density of 10 A/g (Fig. 6c) shows that the capacitor can be charged to saturation in several seconds and that the voltage reached can be 0.8 V. The potential drop in the region of 0.5–0.8 V of the galvanostatic CD curves is attributed to the internal resistance caused by the solid-solid interface between the electrodes and the H_2_SO_4_-PVA gel membrane. The potential drop is generally due to internal resistance mainly from electrical processes at interfaces^47^, and so it is usually larger with solid electrolytes^7^. Figure 6d shows the specific capacitance of the capacitor versus discharge current density. The discharging capacitance values are 68, 62, 60, 58 and 55 F/g (relative to the mass of NG-PAA/PANI) at 1, 2, 4, 5 and 10 A/g, respectively.

Table 2 compares our supercapacitor with previously reported similar PANI based composite electrodes and flexible capacitors. The 68 F/g reported here is more than 13 times the capacitance of a previously reported similar device using composites containing 60 wt.% PANI from mechanically mixing PANI and PAA (5.1 F/g)^20^. The capacitance is more than four times that for a flexible supercapacitor based on PANI-carbon nanotubes composite (PANI-CNT-1) prepared from mixing in organic reagents (16 F/g)^31^, double that from an *in situ* chemical polymerization (PANI-CNT-2) (31.4 F/g)^7^, and still higher than the capacitance of capacitors made from PANI-activated carbon fiber composites (52 F/g)^46^. More importantly, we reach 55 F/g at a high current density of 10 A/g, which is still 80.9% of the capacitance at 1 A/g. The excellent rate capability is promising for applications needing rapid charging-discharging. The energy density of the capacitor is 5.8 Wh/kg at a power density of 1.1 kW/kg, which is higher than observed with flexible supercapacitors based on composites of PANI and carbon nanotubes (0.5 Wh/kg at 0.3 kW/kg)^31^. At a higher power density of 3.9 kW/kg, the energy density is still 5.1 Wh/kg. Long-term cycling performance of the device was evaluated at the constant current density of 1 A/g. The supercapacitor exhibits excellent stability with 83.2% retention of the initial capacitance after 2000 cycles (Fig. 6e). The capacitance is still higher than those devices with PANI based electrode materials^48^,^49^,^50^,^51^. As mentioned, this is largely because of the NG doping, which reduces the swelling of PANI when charging; without this improvement, the redox sites in the polymer backbone do not survive the swelling-shrinking over many charging cycles. There are no significant changes in the CVs when bending the capacitor at angles from 0 to 135° at a scan rate of 50 mV/s (see Fig. 6f).

## Conclusion

We have developed a novel NG-PAA/PANI composite via a preparation that separates *in-situ* polymerization and subsequent mixing with irregularly shaped PANI nanoparticles, which allows a very high PANI content of up to 69 wt.% without compromising the processibility of the material. The PANI weight content was optimized for defect-free coverings on CC carbon fibers. Optimizing NG doping obtains a superior weight specific capacity. All-solid-state capacitors with NG-PAA/PANI composites containing 20% PANI on CC reach 68 F/g at 1 A/g, a thirteen-fold improvement over the previously reported PANI/PAA capacitors, and more than four times larger than flexible capacitors based on PANI-carbon nanotube composites. The electrochemical properties and stability are retained under bending angles up to 135^o^. The NG-PAA/PANI used here contained only 20% PANI. Using instead the optimal 32% PANI is expected to lead to even better performance parameters, which we leave to future research.

## Methods and Experimental Section

### Materials

Polyacrylic acid (PAA) (average molecular weight ~450,000 Da), Aniline, poly(vinyl alcohol) (PVA), ammonium persulphate (APS, (NH_4_)_2_S_2_O_8_), concentrated hydrochloric acid (HCl), and ethylenediamine (EDA) were purchased from Sigma-Aldrich. All chemicals were analytical grade. Aniline was purified by vacuum distillation prior to use. Deionized water with resistivity exceeding 18.0 MΩ cm from a *JL*-RO 100 Millipore-*Q* Plus purifier was used throughout the experiments. 360 μm thick CC (W0S1002) of 12.5 mg/cm^2^ was purchased from CeTech CO., Ltd., China. The CC was immersed in acetone at r.t for 1 h and ultrasound cleaned in ethanol and then water. The washed CC was immersed in 5% KMnO_4_ solution for 0.5 h and rinsed with ethanol and water before air drying.

### Synthesis of PANI nanoparticles

Dissolving 2.28 g APS into 100 ml of 1 M aqueous HCl solution obtained 0.1 M APS solution. Under a vigorous stir below 5 °C, 1 ml of distilled aniline was added drop-wise over 30 min. The solution was stirred for another 2 h and kept in an air tight flask for 12 h at room temperature (r.t.). A dark bluish gray precipitate was collected by filtration, washed with methanol and 0.1 M HCl, and dried at 80 °C in vacuum. The obtained powder was stored in a desiccator.

### Synthesis of PAA/PANI suspensions

PAA/PANI solution was synthesized by an *in-situ* oxidative polymerization as the first step. We typically dissolved 0.9 g PAA and 3.25 g APS into 100 ml of 1 M HCl in a 250 ml conical flask while stirring at 0~5 °C, resulting in 0.05 mM PAA and 14 mM APS. Then, 1 ml of distilled aniline was again added drop-wise over 30 minutes while stirring vigorously below 5 °C. Obtaining a stable aqueous mixture needs stirring for 6 h. The stir was continued for another 24 h at r.t. in order to assure complete polymerization. A dialysis membrane with a molecular weight cut off of ~3500 Da was used to remove PANI oligomer and impurities from oxidative polymerization. Dialysis in 0.1 M HCl continued until the solution became colorless again.

### Synthesis of NG-PAA/PANI composites

NG was synthesized hydrothermally by reducing graphene oxide (GO) from a modified Hummers method as described previously^52^. 500 μL ethylenediamine were added slowly (15 min) into 2 mg/ml stirred GO suspension. The mixture was kept for 12 h in an 100 ml Teflon-lined autoclave in an oven at 160 °C. The products were purified by centrifugation and freeze dried for 7 h. Dried NG power was stirred vigorously into the PAA/PANI suspension, obtaining various graphene contents from 0.05 to 5.00 wt.% (relative to dried product). In order to improve the dispersion in water, an ultrasonic pulse system treated the NG nanosheet in PAA/PANI suspensions with an ultrasonic power of 500 W (pulses last for 9 s with a pause interval of 2 s) for 1 h.

### Characterizations

The weight percentage of PANI in the PAA-PANI suspension before addition of PANI nanoparticles was determined by monitoring the UV-Vis band at 390–410 nm using UV-3600 spectrophotometer of Shimadzu^34^. Fourier-transform infrared (FTIR) spectra of KBr powder-pressed pellets of the NG-PAA/PANI composites were recorded on a Spectrum-GX spectrometer of Perkin Elmer. X-ray diffraction (XRD) was performed with an Ultima-III X-ray diffractometer. Morphology was investigated using scanning electron microscopy (SEM) (Hitachi S-4800) and transmission electron microscopy (TEM) on a JEOL JEM-2100 electron microscope.

### Electrochemical performance

Measurements were carried out using a computer controlled CH1660D electrochemical workstation. To obtain composite covered working electrodes for the NG optimization, 200 μl of NG-PAA/PANI composite suspension was cast onto Pt foils before drying at 60 ^o^C for 50 min. CC was instead dipped into the suspension and dried in air at r.t. for 12 h. All substrate areas are 4 cm × 1 cm, but the effective area is 3 cm^2^ after reserving 1 cm for connection clamps. The mass loading after drying is ~2 mg for Pt, but 4 mg for the CC. The conductivity of the composite film was measured after pouring onto silicon and using a four-probe assembly connected with KEITHLEY nanovoltmeter instrument (model 2400). Cyclic voltammetry (CV) and galvanostatic charge-discharge (CD) measurements were conducted in a three-electrode cell where a Pt electrode serves as the counter electrode and a standard Ag/AgCl electrode as reference. The electrolyte was 1 M H_2_SO_4_ aqueous solution. CVs measurements were performed between −0.2 and 1.0 V (vs. Ag/AgCl). Specific capacitances were calculated from the galvanostatic CD curves via *C*_s_ = (*I*Δ*t*)/(*m*Δ*V*), where *I* is current, Δ*t* is the discharge time, *m* the mass of active material, and Δ*V* the voltage change due to a discharge.

### Fabrication of flexible solid-state carbon cloth supercapacitors

Solid gel electrolyte was prepared by pouring 10 g of concentrated H_2_SO_4 _into 100 ml of deionized water and adding 9 g of PVA powder. The mixture was continuously stirred and heated to 89 ^o^C until it became clear. A subsequent 2 h slow stir removes bubbles. The resulting solution was transferred into a mold to obtain the H_2_SO_4_-PVA gel membranes. To assemble capacitors, two impregnated CC were used as the electrodes. The ~0.1 mm thick H_2_SO_4_-PVA gel membrane was sandwiched in between and thus serves as a solid electrolyte and separator dielectric. Short pieces of copper foils pressed on the ends of the CC served as connectors.

## Additional Information

**How to cite this article**: Wang, Y. *et al.* High-Performance Flexible Solid-State Carbon Cloth Supercapacitors Based on Highly Processible N-Graphene Doped Polyacrylic Acid/Polyaniline Composites. *Sci. Rep.* **6**, 12883; doi: 10.1038/srep12883 (2016).

## Supplementary Material

Supplementary Information

## Figures and Tables

**Table 1:** *C*_PAA_ versus specific capacitance and conductivity of PANI-PAA composite films (without addition of PANI nanoparticles or NG).

*C*_PAA_/mM	Absorption max/nm	Capacitance/F g^−1^	Conductivity/S cm^−1^
0.01	430	/	/
0.02	404	145.83	0.077
0.03	398	94.48	0.039
0.04	394	46.68	0.035

**Table 2:** Performance of the supercapacitor based on NG-PAA/PANI with 20% PANI and some previously reported PANI based composite electrodes and flexible capacitors.

Composites	Potential	Electrode	Capacitor	Retention	Reference
NG-PAA/PANI	0 ~ 0.8 V	521 F g^−1^	68 F g^−1^	83.2%	This work
PAA-PANI	0 ~ 1.0 V	/	5.1 F g^−1^	/	[20]
PANI-CNT-1	0 ~ 1.0 V	/	16 F g^−1^	50%	[31]
PANI-CNT-2	0 ~ 0.8 V	312 F g^−1^	31.4 F g^−1^	95.2%	[7]
PANI-ACF	0 ~ 1.6 V	240 F g^−1^	52 F g^−1^	80%	[46]
PANI-CNF	0 ~ 1.0 V	220 F g^−1^	/	20%	[48]
PANI-PVA	0 ~ 1.0 V	106 F g^−1^	/	/	[49]
PANI-GO	−0.2 ~ 0.8 V	162 F g^−1^	/	87.9%	[50]
PANI-RGO	0 ~ 0.8 V	243 F g^−1^	/	86%	[51]

CNT: carbon nanotube, ACF: activated carbon fiber, CNF: carbon nanofiber.
