# Supplementary material for: High-Performance Flexible Solid-State Carbon Cloth Supercapacitors Based on Highly Processible N-Graphene Doped Polyacrylic Acid/Polyaniline Composites
Source: Sci Rep. 2016 Feb 17;6:12883. doi: 10.1038/srep12883 (PMC4756666; doi:10.1038/srep12883)
Supplement: Supplementary Information [file srep12883-s1.doc]

**Electronic Supplementary Information**

**High-Performance Flexible Solid-State Carbon Cloth Supercapacitors Based on Highly Processible N-Graphene Doped Polyacrylic Acid/Polyaniline Composites**

**Yongguang Wang, Shaochun Tang*****, Sascha Vongehr, Junaid Ali, Xiangyu Wang & Xiangkang Meng***

*Institute of Materials Engineering, National Laboratory of Solid State Microstructures, and College of Engineering Applied Sciences, Nanjing University, Jiangsu, P. R. China*

**Corresponding authors**

* Tel.: (+86)-25-83685585; fax: (+86)-25-83595535;

Correspondence and requests for materials should be addressed to S.C. Tang ([tangsc@nju.edu.cn](mailto:tangsc@nju.edu.cn)) or X.K. Meng ([mengxk@nju.edu.cn](mailto:mengxk@nju.edu.cn)).

**Figure captions:**

**Fig. S1.** UV-Vis absorption spectra of PAA/PANI suspensions obtained from the polymerization reactions with different *C*_PAA_.

**Fig. S2.** SEM images showing the surface of PAA/PANI films with different *w*_PANI_ of (a) 25, (b) 28, (c) 37, (d) 42, (e) 56, and (f) 69 wt.%.

**Fig. S3.** CV curves of Pt electrodes coated with the PAA/PANI composite film containing a high PANI percentage of 42 wt.%.

**Fig. S4.** (a) XRD patterns and (b) FT-IR spectra of the GO precursor and resulting NG, (c) XPS survey and (d) high resolution N1*s* XPS spectra of NG.

**Fig. S5.** CV curves of pure CC substrate and CC with the optimal NG-PAA/PANI composite.

**Fig. S6.** (a) Schematic illustration to the all-solid-state capacitor using flexible copper foil as substrate (optical image below), and (b) its CV curves at different scan rates from 5 to 100 mV/s, and (c) its CD curves at different current densities.


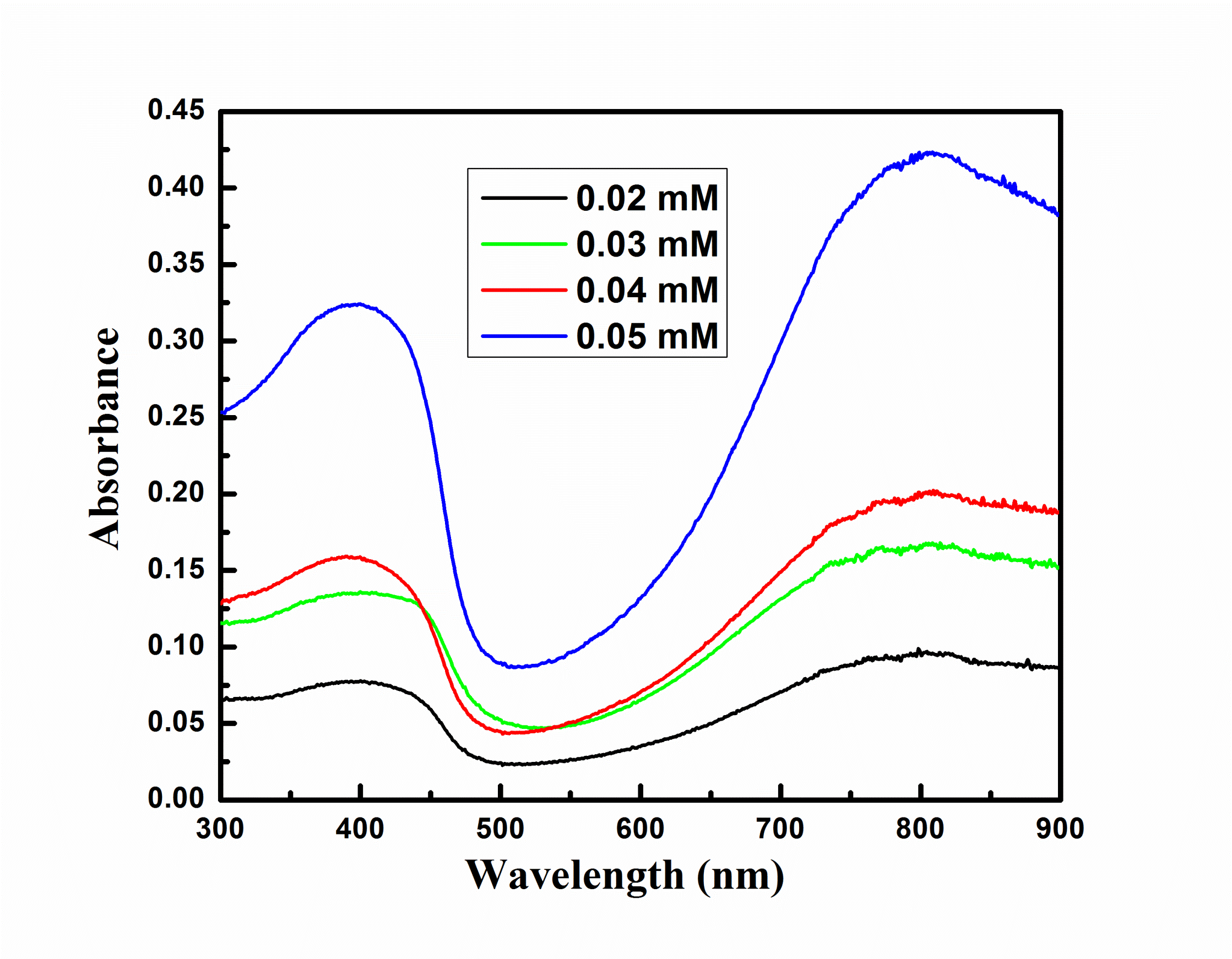


**Fig. S1.** UV-Vis absorption spectra of PAA/PANI suspensions obtained from the polymerization reactions with different *C*_PAA_.


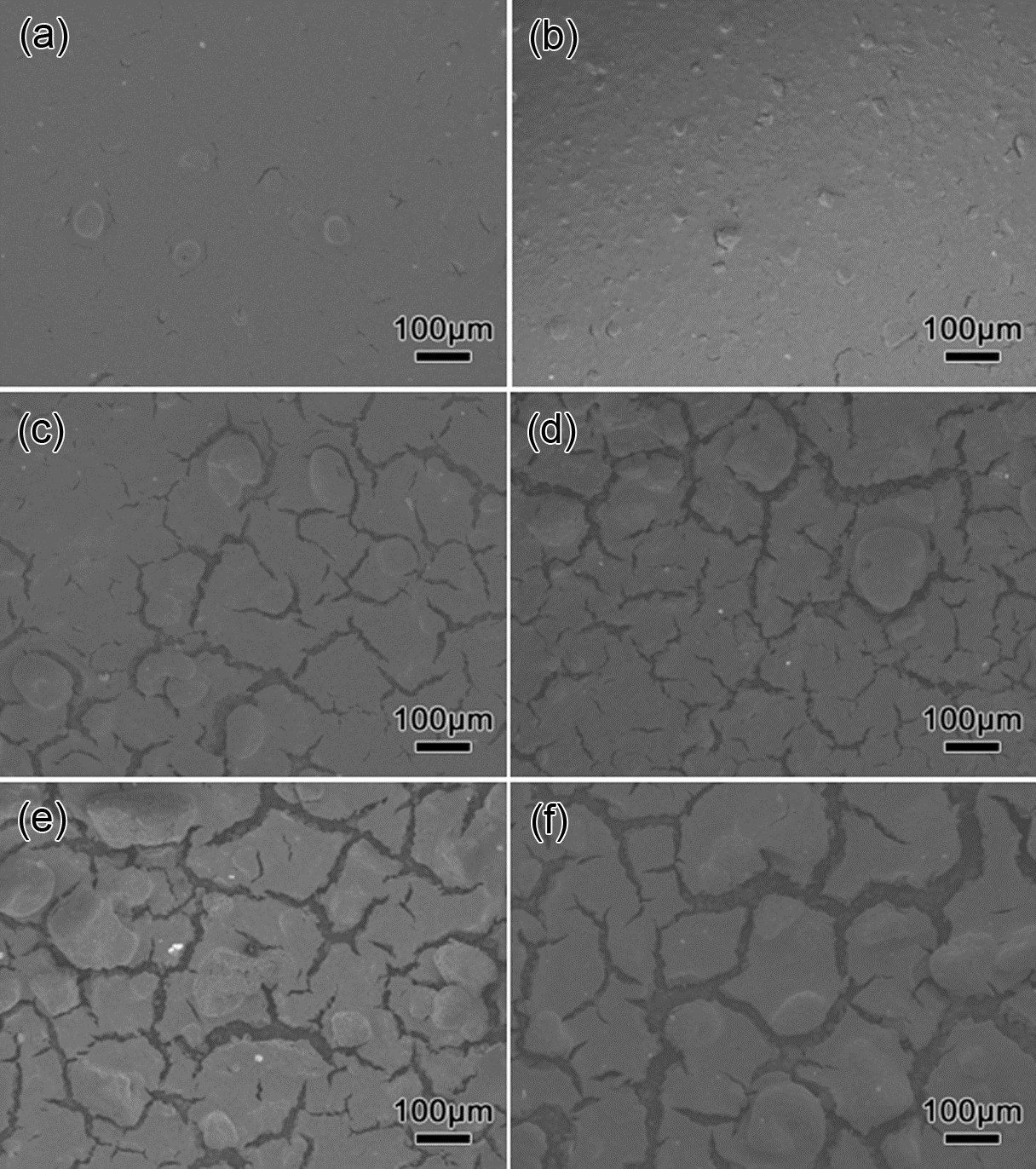


**Fig. S2.** SEM images showing the surface of PAA/PANI films with different *w*_PANI_ of (a) 25, (b) 28, (c) 37, (d) 42, (e) 56, and (f) 69 wt.%.


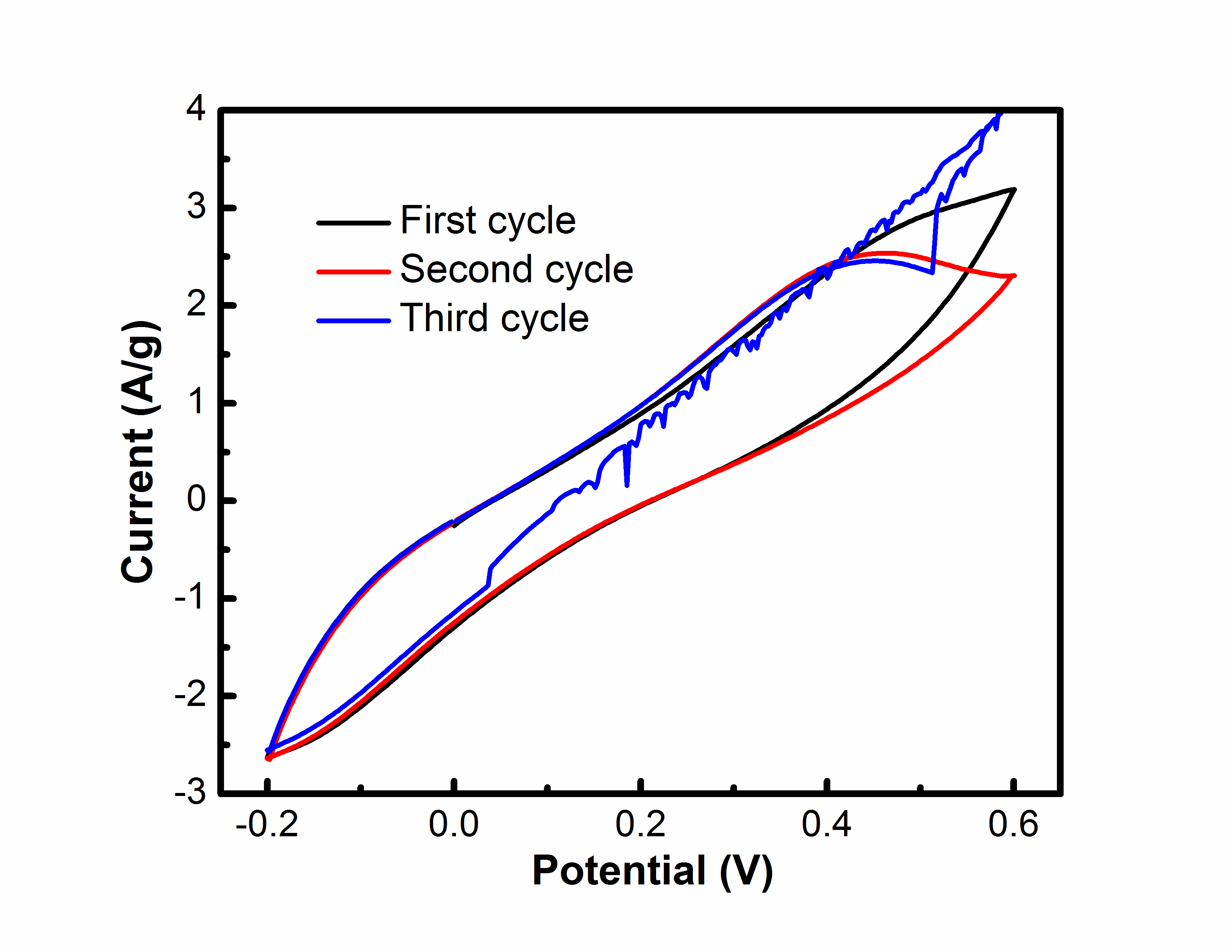


**Fig. S3.** CV curves of Pt electrodes coated with the PAA/PANI composite film containing a high PANI percentage of 42 wt.%.


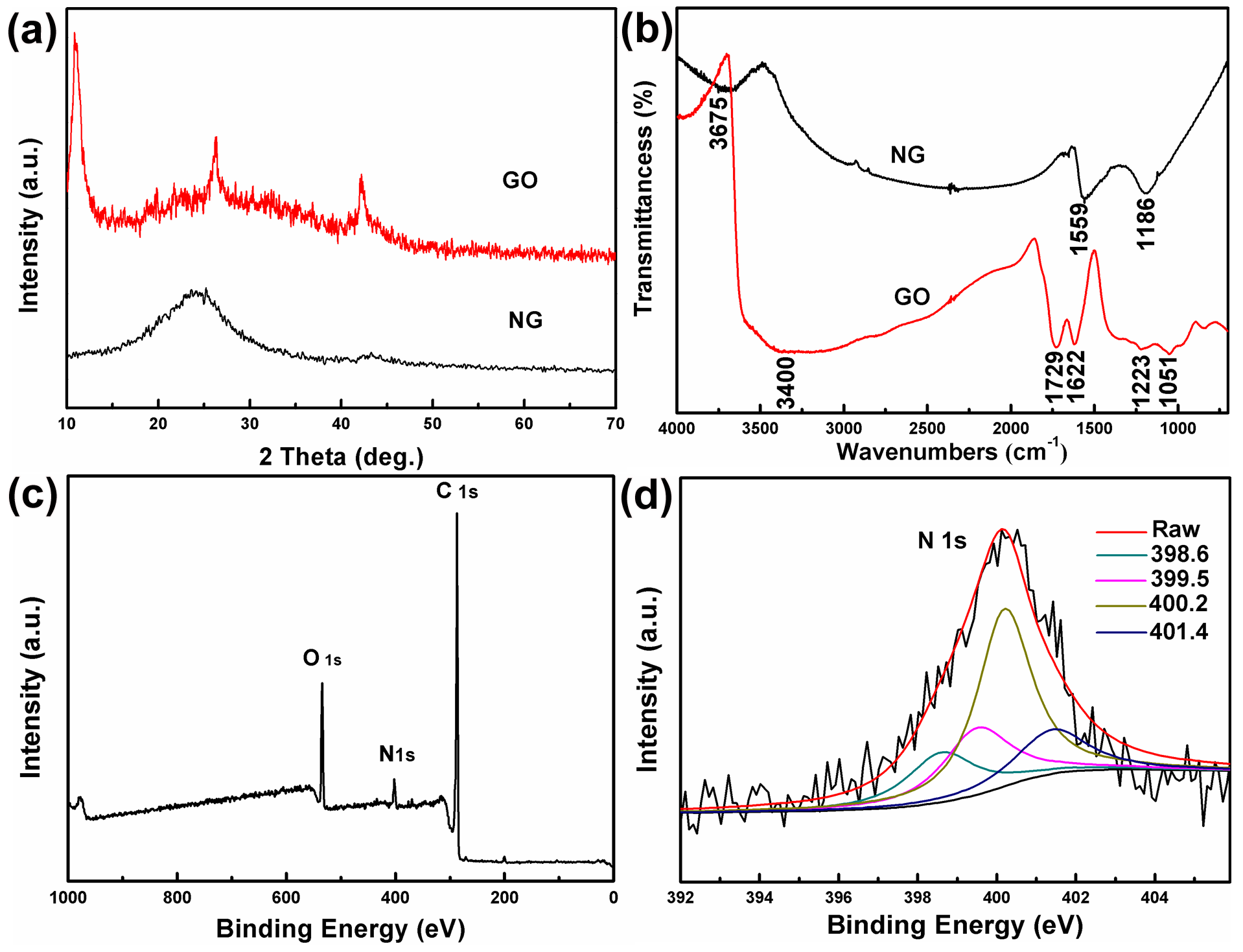


**Fig. S4.** (a) XRD patterns and (b) FT-IR spectra of the GO precursor and resulting NG, (c) XPS survey and (d) high resolution N1*s* XPS spectra of NG.

Fig. S4a shows XRD patterns of GO (red curve) and resulting NG (black curve). A broad and weak diffraction is observed at about 26.4^o^ and assigned to the (002) of graphene. The inter-layer spacing of NG is calculated to be 3.42 Å, which is much lower than that of GO (8.02 Å), but slightly higher than that of natural graphite (3.37 Å). The characteristic GO peak at 11.03^o^ is not visible, indicating complete conversion. No peaks from other phases are present. Fig. S4b shows FT-IR spectra of GO and NG. The O-H and C=N functional groups are obvious. A notable C=N stretching vibration mode occurs near 1559 cm^-1^, resulting from the NG. XPS is often utilized to investigate the elemental surface composition of samples and the corresponding valence states. The XPS survey (Fig. S4c) shows anticipated binding energy positions, corresponding to C, O, and N elements. In the high-resolution N1s spectrum (Fig. S4d), four main kinds of doped nitrogen were observed, including pyridinic nitrogen (398.6 eV), amine moieties or other nitrogen bonds (399.5 eV), pyrrolic nitrogen (400.2 eV), and protonated N or the graphitic N (401.4 eV).


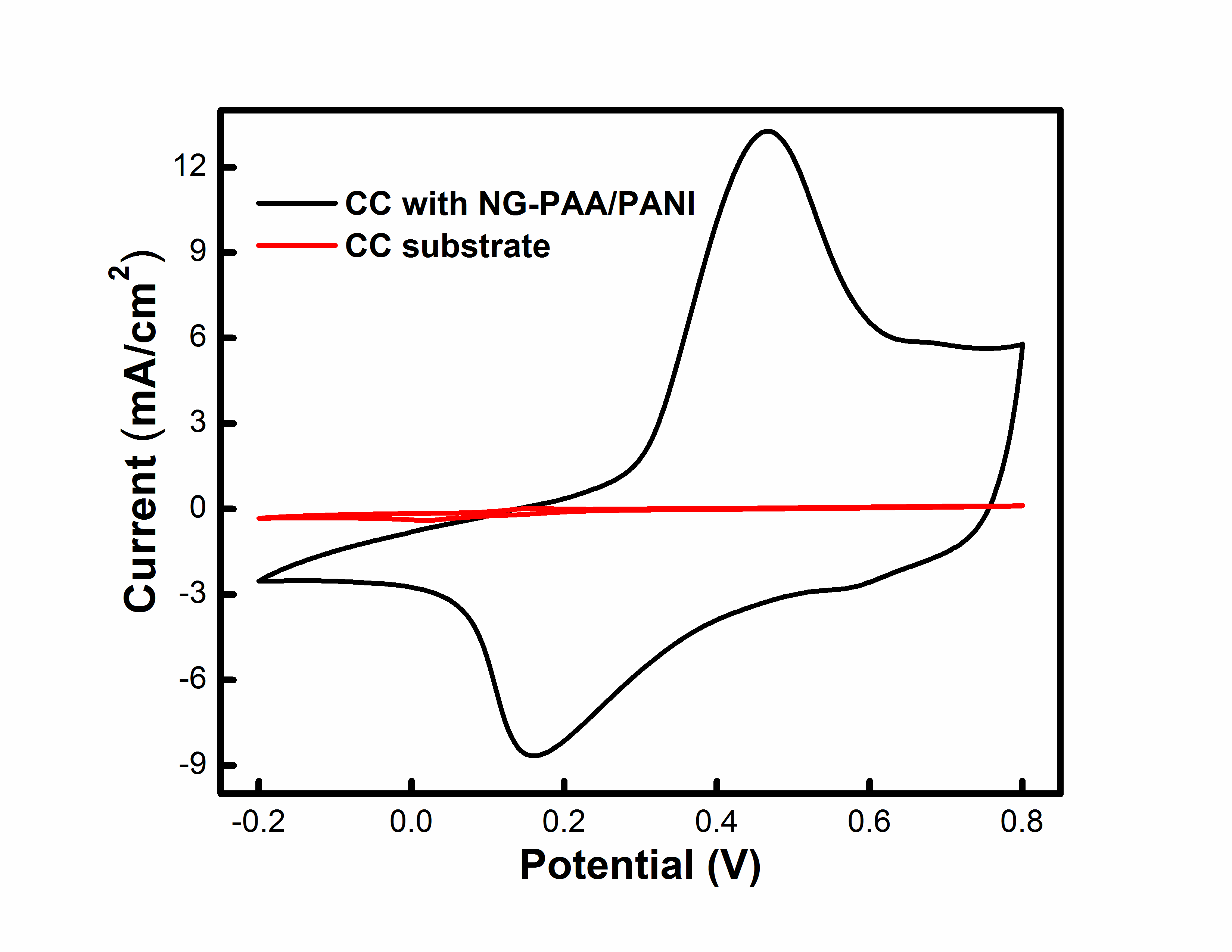


**Fig. S5.** CV curves of pure CC substrate and CC with the optimal NG-PAA/PANI composite.


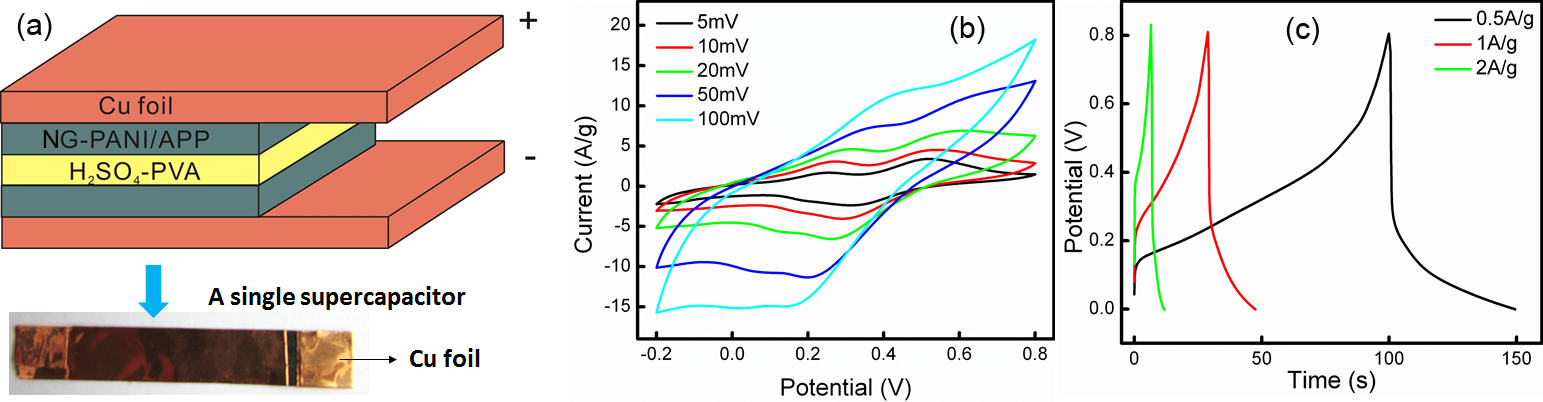


**Fig. S6.** (a) Schematic illustration to the all-solid-state capacitor using flexible copper foil as substrate (optical image below), and (b) its CV curves at different scan rates from 5 to 100 mV/s, and (c) its CD curves at different current densities.

Using the NG-PAA/PANI composite films on Cu foils, flexible all-solid-state capacitors with H_2_SO_4_-PVA as electrolyte were assembled. Fig. S6a shows its sandwich structure, and a supercapacitor is shown in the lower inset. CV measurements (Fig. S6b) show a pseudocapacitive behavior and the curves’ shape is kept even at high scan rates. There is no change in the curves’ shape under bending, which promises a great potential in flexible devices. The specific capacitances of the capacitor under various bending angles are almost constant, which confirms the potential applications of NG-PAA/PANI composite films as flat, flexible devices. The CD curves (Fig. S6c) indicate that the capacitor can be charged to saturation within several seconds at 2 A/g, and that the voltage can be up to 0.8 V. The fast response confirms the efficient ion and electron transport within the films.
